# Influence of fermentation conditions, and the blends of sorghum and carrot pulp supplementation on the nutritional and sensory quality of tef* injera*

**DOI:** 10.1038/s41598-024-62420-5

**Published:** 2024-06-04

**Authors:** Mohammed Suraj, Mikiyas Abewaa, Ashagrie Mengistu, Geremew Bultosa, Nigussie Bussa

**Affiliations:** 1 Department of Chemical Engineering, College of Engineering and Technology, Wachemo University, Hossana, Ethiopia; 2 The Federal Democratic Republic of Ethiopia, Manufacturing Industry Development Institute, P.O. Box 1180, Addis Ababa, Ethiopia; 3 Department of Food Science and Postharvest Technology, Haramaya University, Haramaya, Ethiopia

**Keywords:** Anti-nutrient factors, Blending ratio, Carrot pulp, Fermentation, Functional foods, *injera*, Chemistry, Engineering

## Abstract

*Tef *[*Eragrostis tef *(Zucc.) Trotter], an ancient cereal primarily grown in Ethiopia, is becoming increasingly popular worldwide due to its high iron content and gluten-free nature. However, it has been reported that *injera* produced only with tef flour lack certain vital nutrients. Therefore, this specific study was conducted to supplement tef *injera* with other food materials of better nutritional value and compensate its expensive market price with sorghum cereal flour. The effect of fermentation conditions, and the sorghum and carrot pulp blending ratio on the nutritional value and sensory quality of tef *injera* was investigated. The factorial approach of the experimental design was conducted considering the nutritional value and sensory quality of the *injera* made of three main blending ratios of tef, sorghum, and carrot (60% tef: 30% sorghum: 10% carrot pulp, 45% tef: 45% sorghum: 10% carrot pulp and 30% tef: 60% sorghum: 10% carrot pulp) as experiential variables. The raw materials and *injera* were characterised for their proximate composition, physicochemical property, mineral composition, microbial analysis, and sensory attributes, using standard methods. The results of the study show that fermentation conditions and blending ratios have a significant effect on the nutritional, anti-nutritional, mineral content, microbial quality, and sensory properties of blended *injera* products, where higher values of ash, crude protein, crude fat, Total titratable acidity (TTA), Fe, Zn, and Ca (2.30%, 11.34%, 2.62%, 3.53, 32.97 mg/100 g, 2.98 mg/100 g and 176.85 mg/100 g, respectively) were analyzed for the co-fermented *injera* sample. In addition, a lower microbial count was observed in co-fermented *injera* samples, whereas microbial counts in injera samples prepared from carrot pulp-supplemented dough after the co-fermentation of tef and sorghum flours were observed to be higher. The *injera* product made using blending ratio of 60% tef: 30%sorghum: 10% carrot co-fermented was found to be the optimum result due to its very good nutritional improvement (i.e., reduction of some anti-nutritional factors, microbial contents, pH and increased contents of some minerals, crude protein, crude fat, TTA and improved most of the sensory quality of the supplemented *injera* product). According to this study, sorghum and carrot supplementation on tef could improve the nutritional value of *injera* while also providing an instant remedy for the growing price of tef.

## Introduction

In Ethiopia, there are varieties of tef breeds in terms of both color and mineral content, of which Grain tef [*Eragrostis tef* (Zucc.) *Trotter*] is recognized for its better nutritional value than common cereal grains. Due to this quality, it is consumed as a whole grain, mostly as an *injera* (a fermented, spongy, sour, circular flat bread)^1,2^. Furthermore, its being free from the type of gluten found in other types of cereals such as wheat makes it highly preferable and widely consumed by people with severe allergies to wheat gluten^2–4^. A number of studies show that tef grain *injera* is superior among other cereal grains. However, its alarmingly increasing cost necessitates a search for other food items such as sorghum. Sorghum (the most important staple crop in Africa and uniquely adapted to the semiarid and subtropical climatic conditions of the continent) is potentially suitable for use in composite flours^5–7^, and it has a definite advantage over maize and other tropical cereals in composite flours because of its bland flavor and white color in tan plant types^8^. However, due to its high starch gelatinization temperature and low water-holding capacity, sorghum flour tends to give a drier, grittier, and firmer texture to breads and biscuits made with sorghum and wheat composite flours^9,10^. Preparing *injera* from sorghum has considerable economic benefits over tef due to its much lower price. However, the problem is that sorghum *injera* rapidly becomes firm and friable upon storage. It was reported that sorghum cultivar differences existed for *injera-*making quality and staling. It has also been found that the use of composite flours of sorghum and tef improved *the texture of the injera* compared with 100% sorghum^11^. Researchers conducted a consumer preference sensory test of *injera* from different cereals and reported that sorghum was accepted as a substitute for tef *injera*^12–14^. Additionally, the development of bakery products containing carrot pulp could have potential health benefits due to its high carotenoid content.

Epidemiological and clinical investigations have associated diets rich in fruits and vegetables with a reduced risk of heart, cardiovascular, neurological, and chronic diseases and various forms of cancer^15^. Vitamin A deficiency is the most common dietary deficiency disorder. For this reason, World Health Organization (WHO), Food and Agriculture Organization of the United Nations (FAO), United States Department of Agriculture (USDA) and European Food Safety Authority (EFSA) recommend increased fruit and vegetable consumption^16^. Vegetables are typically good sources of vitamins, particularly vitamins A and C, and of minerals, especially potassium, magnesium, calcium, and iron. Further processing of vegetables provides a range of useful materials, including sugar and starch, which are added to other food products^17^. Fruit and vegetable juices have become important in recent years due to the overall increase in natural juice consumption as an alternative to traditional caffeine-containing beverages such as coffee, tea, or carbonated soft drinks^18,19^. Carrot contains not only nutritional antioxidants such as vitamins A, C, and E but also a great quantity of non -nutritional antioxidants, such as carotenoids, flavonoids, flavones, and other phenolic compounds^20,21^. It is mainly consumed as a raw drink, converted to a juice drink, used as a salad, cooked as a vegetable dish, and used to make sweet dishes.

In Ethiopia, there are various traditional foods with enormous potential to be developed for functional foods for the benefits of Ethiopian consumers and for competitive functional food markets. Sorghum and vegetables such as carrots are the cheapest sources of calories for both human nutrition and animal feeding. Additionally, in the current situation of Ethiopia, buying pure tef for household consumption becomes very difficult. To minimize the cost of making pure tef *injera*, mixing sorghum with some portion of tef is currently a common practice in most parts of Ethiopia. This is normally in combination with other cereals (bread wheat, maize or millet flours), but scientific study on its proportion, composition and other attributes is needed. In most parts of Ethiopia, carrots are mainly consumed raw, rarely converted to juice drinks, used as salads and cooked as vegetable dishes. It is an important fact that cereal grains tend to be limited in some vitamins, whereas vegetables are rich in these vitamins. Compositing grain with carrot pulp may improve the content of vitamin-A in cereal grain products. Thus far, it seems there is no research done on the characterization of tef *injera* that are supplemented with carrot pulp and sorghum at the same time. Therefore, in this study, carrot pulp was supplemented into a mixture of sorghum and tef flour before fermentation (co-fermentation) and after fermentation (into fermented tef and sorghum batter) for use as a staple food. Hence, this study was conducted to evaluate the influence of sorghum and carrot pulp supplementation on the chemical composition, microbial load, and sensory quality of tef *injera.*

## Material and methods

### Raw material preparation

**Figure 1:**
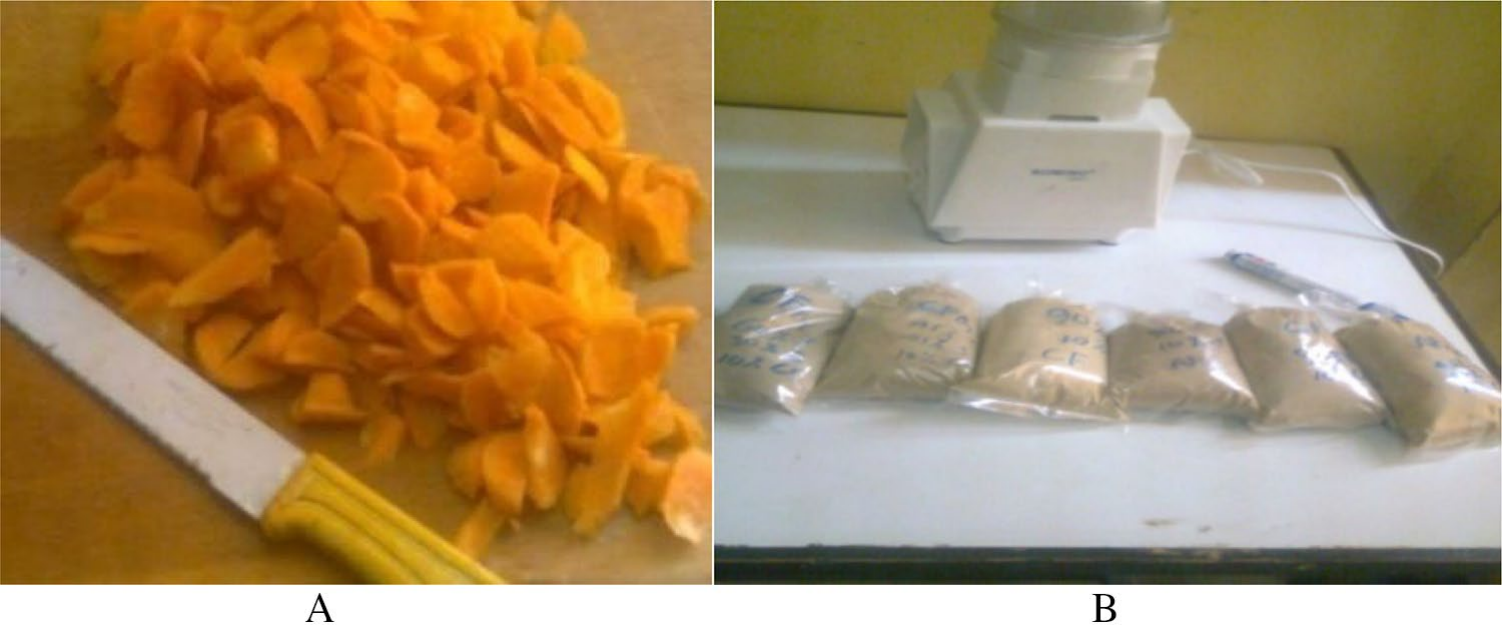
Sample preparation: sliced carrot (**A**), milled sample (**B**).

Tef (*Eragrotis tef*), sorghum (*sorghum bicolour*), and carrot (*daucus carota*) samples for the investigation purpose were procured from the Debre Zeit Agricultural Research Centre, Awash Melikasa Agricultural Research Centre, and Harar local market, respectively. The collection of plant material was supported by relevant institutional, national, and international guidelines and legislation. The cleaning of sorghum and tef grains from impurities and broken seeds was performed manually, and the grains were then milled into fine flour using the stone milling process (a traditional cottage grain milling system) and kept in polythene bags at 4 °C for a week, as shown Fig. 1. The carotene-type carrot with pigment (Nantes variety) commonly grown in Ethiopia was used for this study, and the fresh root harvested at optimum maturity (90 days) was obtained from Harar's local market. The non-edible portion of the carrot was removed, and then the root was washed with tap water to remove the soil and air-dried before it was stored in a refrigerator at 4 °C for 2 days^22^. The clean carrot taken from the refrigerator was cut manually into thin slices of 5 ± 1 mm thickness using stainless steel knives to create a suitable condition for effectively reducing its size^23^. Then, the whole raw carrot was mashed after peeling to reduce the size without extraction of juice using a juice blender (Vertical cutter-mixer, France).

### Experimental design

Supplementation of sorghum and carrot pulp with tef *injera* was carried out considering two scenarios where the carrot pulp was supplemented as co-fermentation (CF) and after sorghum and tef fermentation (AF) were carried out. For both cases, three formulations were designed. Blending ratios are (${B}_{1}$ = 60% tef flour: 30% sorghum flour: 10% carrot pulp), (${B}_{2}$ = 45% tef flour: 45% sorghum flour: 10% carrot pulp) and (${B}_{3}$ = 30% tef flour: 60% sorghum flour: 10% carrot pulp) with three replication where B_1_, B_2_ and B_3_ indicate blending ratios of 1, 2 and 3, respectively. All experiments were carried out in triplicate, and the results are provided as the mean plus standard deviation. Finally, this design was used to investigate the effect of fermentation conditions and blending ratios on *injera* quality.

### *Injera* making process

**Figure 2:**
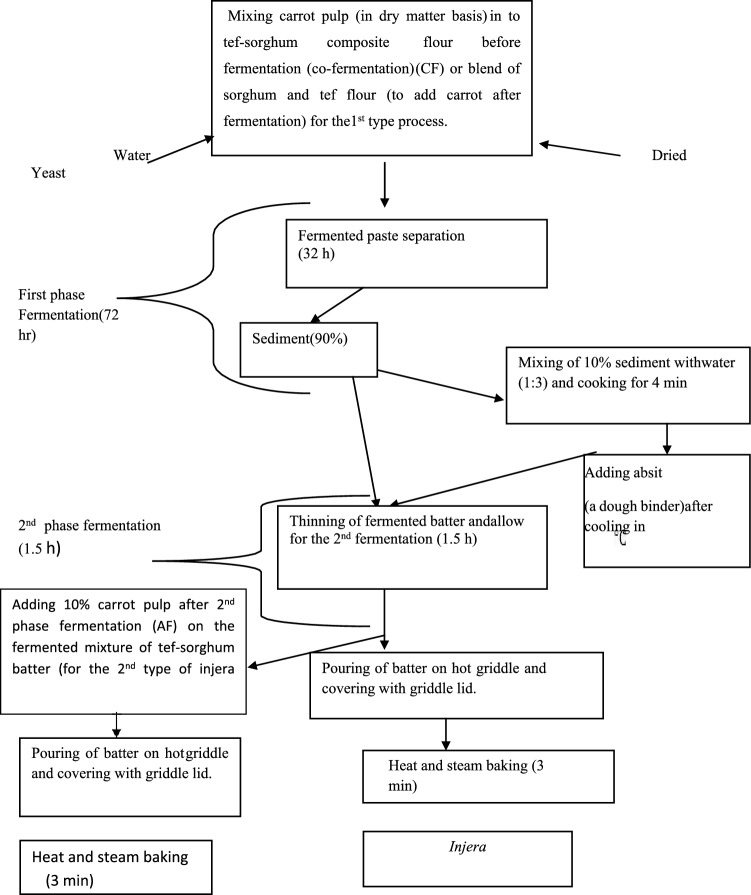
*Injera* preparation process.

A standard recipe for making Tef *injera* containing sorghum and carrot pulp was used. The process involves fermentation and then baking the butter using a method of the *injera*-making process^24^. The tef-sorghum flour-carrot pulp composite was mixed and co-fermented using 2 L of water. Thereafter, the dough was kneaded by hand to attain optimum consistency, and after adding dry yeast, the dough was fermented at room temperature for ≈ 72 h. After the completion of the fermentation process, 10% of the fermented dough was mixed with 1:3 water and then boiled in boiling water for 4 min. The boiled batter was then cooled to 46 °C and returned to the fermenting dough. After thorough mixing, the batter was fermented at room temperature for 2 h, and additional water was added to the fermented dough to achieve optimum batter consistency. Finally, the fermented batter was poured circularly on a hot clay griddle, covered with a lid to prevent steam from escaping, and baked for 3 min. The second type of *injera*-making process followed the same steps, but the only difference was adding carrot pulp to fermented sorghum and tef mixture batter after the second fermentation (AF), as shown in Fig. 2.

### Raw material and product characterization

#### Chemical composition of the raw materials and dried *injera* product

The proximate analysis of tef, carrot, sorghum, and *injera* was carried out following the protocols of the Association of Official Analytical Chemists (AOAC)^29^, as shown in Table 1. In addition, gross energy was determined by calculating the individual fat, carbohydrate, and protein contents, then using Atwater’s conversion factors of 16.7 kJ/g (4 kcal/g) for protein, 37.4 kJ/g (9 kcal/g) for fat, and 16.7 kJ/g (4 kcal/g) for carbohydrates, and expressed in calories as shown in Eq. (1)^25^.

1 $${\text{E}} = \left[ {{9} \times {\text{Lipids }}\left( \% \right) + {4} \times {\text{Protein }}\left( \% \right) + {4} \times {\text{Carbohydrates}}\left( \% \right)} \right]$$

**Table 1:** Raw materials and product proximate compositions.

S/no	Analysis	Method
1	Moisture content	AOAC 925.10
2	Ash content	AOAC 923.03
3	Protein content	AOAC 979.09
4	Crude fiber	AOAC 962.09
5	Fat content	AOAC 920.39
6	Carbohydrate content	100-(% moisture + %fiber + % ash + %protein)

#### Analysis of pH and titrable acidity of the baked *injera*

The pH of the *injera* was determined by direct reading using a glass electrode attached to a pH meter (EUTECH Instruments pH 510, Malaysia), following AOAC official method 945.42. The titratable acidity of the *injera* sample was determined by titrating a volume of an aliquot of injera filtrate with 0.1 N NaOH, using a 1% phenolphthalein end point as the indicator. Finally, the percentage titrable acidity of the baked *injera* was determined using Eq. (2)^26^.

2 $${\text{Titratable}}\;{\text{acidity }}\left( \% \right) \, = \frac{Vb \times Nb \times 0.009}{{Wb}} \times 100$$

where *Vb* is the volume of the base used, 0.009 is the acid equivalent factor for lactic acid, and *Wb* is the sample weight.

### Determination of anti-nutritional factors

#### Determination of condensed tannin content

The vanillin-HCl method was used to analyze condensed tannins using a modified vanillin-HCl methanol method^27^.The reagent was prepared by mixing 8% concentrated HCl in methanol and 1% vanillin in methanol. The ground sample was placed in a conical flask and then centrifuged and pipetted into a test tube. The absorbance at 450 nm was measured using UV–VIS spectrophotometer (Jenway 6505, U.K), and a standard curve was prepared from catechin. Tannin content was expressed as catechin equivalent as shown in Eq. (3).

3 $${\text{Tannin}}\left( \% \right) = \frac{C \times 10 \times 100}{{200}}$$

#### Phytic acid determination

Phytate-phosphorus (Ph-p) analysis was used to determine phytic acid in flour samples. The flour sample was extracted with 3% trichloroacetic acid and centrifuged (Model 1020DE, Ford Airfield Industrial Estate, UK). The supernatant was used for phytate estimation. The precipitate was digested with concentrated H_2_SO_4_ and H_2_O_2_, converting phosphorus into phosphate. Phosphate was analyzed using phosphomolybdate blue absorbance. The p level was then calculated using a spectrophotometer (Jenway 6505, U.K,), and the phytate was estimated as phytate phosphorus. Precisely, the absorbance at 822 nm was read using spectrophotometer (6505 uv/vis spectrophotometer (Jenway 6505, U.K). The absorbance for sample was subtracted from the blank phosphorus level that was estimated from the calibration curve drawn using concentration versus absorbance. Then, phytate was estimated as phytate phosphorus (i.e., phytate = P × 3.55)^28^.

#### Analysis of total carotenoid

The study analyzed total carotenoids in carrot tissue by homogenizing it with an acetone/ethanol solution, filtering it, and washing it with acetone and ethanol. The total carotenoid content of the sample was then measured using a spectrophotometer (Jenway 6505, U.K) at 470 nm, and the quantity of total carotenoids was calculated using Eq. (4)^29^.

4 $$\text{Total carrotenoid }(\text{mg})=\left(\frac{\text{A }\times \text{ V}\times {10}^{4}}{{\text{A}}^{1\text{\%}}\times 100\times \text{G}}\right)$$

where A is the absorbance at 470 nm; V represents the total volume of solution; G is the g of sample; and A^1%^ represents the specific extinction coefficient (2500).

### Determination of ferric ion reducing antioxidant potential (FRAP)

The antioxidant activity of the raw materials and processed *injera* was determined by extracting flour, and mixing it with phosphate buffer, potassium ferricynide, and 10% trichlorlacetic acid (TCA). The absorbance was measured using a UV‒VIS spectrophotometer (Jenway 6505, U.K). A series of six standard solutions of ascorbic acid were prepared and treated as samples. The antioxidant potential was determined against the standard curve, and the FRAP value was expressed as ascorbic acid equivalent (AAE) in µg per g of sample^30^.

### Microbiological analysis

The analyses of the total aerobic plat count, yeast colony count, and coliform counts was performed using the methodology outlined by^31^. Colony–forming units (CFU) per g were used to express the final results. Random samples of *injera* were collected for microbiological investigation.

### Mineral analysis

An atomic absorption spectrophotometer (Model 210 VGP spectrophotometer, Buck Scientific, East Norwalk, CT, USA) was used to measure the content of calcium, iron, and zinc, as reported by reference^31^. Precisely, a 3 g sample was washed, carbonized, and ashed. The residue was dissolved in HCl, filtered, and washed. Thereafter, a standard iron solution was prepared by dissolving pure iron wire in HCl. Both solutions and samples were analyzed using an atomic absorption spectrophotometer (Model BUCK- 210 VGP, U.S.A.) and absorbance was measured at 248.3 nm. The concentration of samples was determined from the absorbance plot against concentration. Likewise, Zink analysis was done as described for the iron analysis. The ash cake was broken up with stirring glass rod and was dissolved in 10 mL of concentrated HCl. The solution was boiled and evaporated nearly to dryness on a steam bath. The residue was re-dissolved quantitatively in 15 mL 1 M HCl. It was filtered through Whatman filter paper into 100 mL volumetric flask. The paper and the residue were washed thoroughly with water and diluted to 100 mL mark. Standard zinc solutions containing 0.1–1.0 mg/L was prepared from Zn metal using 0.1 mol/L of HNO_3_ solution. Both the standard solutions and the samples were analyzed using the atomic absorption spectrophotometer (Model BUCK- 210 VGP, U.S.A.) and absorbance was measured at 213.8 nm (AACC, 2000). Concentration of samples was read from plot of absorbance against µg/mL Zn.

For calcium analysis, the sample was ashed as described for the iron analysis. The ash cake was dissolved in 10 mL of concentrated HCl. The residue was re-dissolved quantitatively in 15 mL 1 M HCl. It was filtered through coarse porosity filter paper into 100 mL volumetric flask. The paper and the residue were washed thoroughly with water and were diluted to 100 mL mark. Lanthanum (La) stock solution was added to make the final dilution 1% La (i.e., 5 mL La solution to 20-mL flask, 15 mL flask). Standard calcium solutions containing 0.1–1.0 mg/L was prepared from CaCO_3_ using 0.1 mol/L of HNO_3_ solution. Both the standard solutions and the samples absorbance was measured at 422.7 nm using the atomic absorption spectrometer (Model BUCK- 210 VGP, U.S.A.).

Phosphorus content was determined using the absorbance of phosphomolybdate blue method. A sample was digested with concentrated sulfuric acid, H_2_O_2_, distilled water, sodium sulfite, (NH_4_)_6_Mo_7_O_24_·4H_2_O, and L-ascorbic acid. The blue color developed after cooling and was measured using a UV–VIS spectrophotometer. A standard solution was prepared from analytical grade K_2_HPO_4_, and a series of six standard solutions were prepared in 30 mL screw-capped test tubes.

### Sensory analysis

Sensory evaluation of *injera* samples was conducted by 50 member judges selected from Haramaya University food science and postharvest technology students and staff, who know sensory analysis. Tef-sorghum–carrot composite *injera* products were evaluated for sensory attributes after 1 h of baking. The baked *injera* was stored in *massob* covered tightly with a polythene bag. The sensory attributes; appearance (i.e., eyes of *injera* and its underneath appearance), flavor, color, odor, sourness, texture, and overall acceptability, were evaluated using a seven-point hedonic scale. Samples of *injera* (100% tef *injera* (control) and composite of tef-sorghum-carrot pulp *injera*) were stored for 5 min in a *massob* by cutting/slicing into a quarter of the whole *injera* with sharp and nit knife to prevent contamination among the different blending ratios of *injera* products. Coded samples from each selected product were arranged in random order on white plates and served to the sensory judges. Just before each test session, orientation was given to the judges on the procedures of the sensory evaluation sheet^32^.

### Statistical analysis

All data were analyzed using two-factor analysis of variance (ANOVA). Duncan’s Multiple Range Test was applied to calculate the level of significance^33^. Mean values were considered at a 95% significance level (*P* < 0.05). The statistical analyses of the data were conducted using statistical analysis system (SAS) version 9.1.3.

## Results and discussion

### Chemical composition and proximate analysis of raw materials

The results for the chemical compositions of the raw materials used to produce *injera* are shown in Table 2. As depicted by the tabulated data*, tef grain*, sorghum flours, and fresh carrot pulp were observed to have moisture contents of 10.02%, 9.49%, and 88.62%, respectively. The moisture content for *tef flour* was obtained to be in agreement with that reported by Do Nascimento et al.^24^, which was in the range of 9.30 to 11.22%. Similarly, the analyzed moisture contents for sorghum flour and carrot pulp were observed to be in agreement with those reported by Aggarwal and Kaur^26^ and Socioeconomics^34^, which were between 8 and 12% and 88.87%, respectively. According to reports by Bultosa^27^ and Idris et al.^28^, the ash contents of tef grain and sorghum were found to be between 1.99% and 3.16% and 1.2 and 1.8, respectively. On the other hand, the determined ash content for carrot pulp was evaluated to be 0.75% greater than that recorded by the United States Department of Agriculture (USDA), which might be due to breed variety and cultivar differences^35^.

**Table 2:** Chemical composition and total carotenoids analyzed for raw materials.

Raw materials	Energy (kcal/100 g)	Moisture (%)	Ash (%)	Crude protein (%)	Crude fat (%)	Crude fiber (%)	COH (%)	Total carotenoid (µg/100 g)
Tef	354.85 ± 0.34^a^	10.02 ± 0.29^a^	2.19 ± 0.11^a^	10.66 ± 0.26^a^	2.33 ± 0.38^b^	1.99 ± 0.00^a^	72.81 ± 0.58^a^	ND
Sorghum	356.08 ± 0.61^b^	9.49 ± 0.34^b^	1.70 ± 0.62^b^	9.93 ± 0.28^a^	2.00 ± 0.12^a^	2.29 ± 0.00^a^	74.59 ± 0.40^b^	ND
Carrot	29.32 ± 0.32^a^	88.62 ± 2.16^a^	1.95 ± 0.07^a^	0.78 ± 0.08^a^	0.24 ± 0.05^a^	2.40 ± 0.36^a^	6.01 ± 1.91^a^	8920.67 ± 9.02^a^

All values are mean ± STDEV on dry basis except moisture (wet basis), *ND* Note detected.

^a,b,c,d,e,f,g^Means ± standard deviations with different letters after data within a column represent differences at 95% probability levels.

The crude protein content results revealed that tef flour has a better protein content (10.66%) than other cereals, such as maize (8.3%), sorghum (7.1%), barley (9.0%), and millet (7.2%), and is almost equivalent to wheat (10.3%)^29^. This result of crude protein content was perceived to be in close agreement with the one reported by Kebede^36^, 10.7% and shown to lie between 9.4 and 13.3%, which is reported by Bultosa^27^. Sorghum flour was found to have a crude protein content in the range of 7.5 to 10.8% of that reported by Idris et al.^28^. However, it was below the range of 11–13% reported by Dendy^37^. This significant variation in sorghum composition might be due to differences in the genetics and environment of cultivation^38^. In this investigation, the crude protein content obtained for carrot pulp was observed to be 0.93% less than that reported by USDA^35^, which might be due to the difference in cultivars and genetic variety.

The results for crude fiber content for tef flour and sorghum flour, 1.99% and 2.29%, respectively. This was in close agreement and as observed to be within the ranges of 2.0 to 6.6% and 2.3% to 2.7% reported by Bultosa^27^ and Idris^28^, respectively. However, the crude fiber content for carrot pulp (2.4%) was evaluated to be 1.2% greater than the value reported by USDA^35^, which could result from variations in cultivating varieties and geographical differences.

The total carbohydrate contents of tef flour were found to be closely in concordance with the one reported by Bultosa and Taylor^39^, 73.0%. Similarly, the result obtained for sorghum flour was observed to lie in the range of 71.4% to 80.7%, as reported by Idris^28^. On the other hand, the total carbohydrate content for carrot pulp was evaluated to be 9.58% less than the value reported by USDA^35^, which is attributed to geographic, environmental, and plant genetic variety.

### Mineral contents of raw materials

The micronutrient analysis results of the *injera* ingredients are shown in Table 3. The iron content for the Qunicho tef variety, 36.36 mg/100 g, determined in this study was determined to be slightly lower than 37.70 mg/100 g reported by Abebe et al.^40^. This insignificant variation was probably attributed to the mineral composition of the soil on which the tef was grown and the mineral anchorage capacity of the tef variety. In a similar manner, the iron content of sorghum, 5.23 mg100 g, was found to be in the 4–5.5 mg/100 g range as reported by Idris^28^^.^ In contrast, the Fe content for carrot pulp, 0.20 mg/100 g, in this specific study was observed to be less than the outcome disclosed by USDA^35^, 0.30 mg/100 g.

**Table 3:** Mineral composition of the raw materials.

Raw material	Fe (mg/100 g)	Zn (mg/100 mg)	Ca (mg/100 mg)	P (mg/100 mg)
Tef	36.36 ± 1.19^a^	1.67 ± 0.25^b^	115.02 ± 3.31^a^	161.57 ± 5.38^a^
Sorghum	5.23 ± 0.69^a^	1.36 ± 0.28^b^	280.83 ± 2.19^a^	174.86 ± 6.74^b^
Carrot	0.20 ± 0.16^b^	0.44 ± 0.10^b^	34.03 ± 1.63^b^	38.14 ± 1.58^a^

*ND* not detected.

^a,b,c,d,e,f,g^Means ± standard deviations with different letters after data within a column represent differences at 95% probability levels.

The Ca content of tef flour, 115.02 mg/100 g, was found to be less than the outcome disclosed by Abebe et al.^40^, 147.00 mg/100 g, but it was seen to be greater than the one reported by Haileslassie et al.^41^, 56 mg/100 g. In contrast, the determined calcium content for sorghum, 280.83 mg/100 g, was seen to lay in the range of 285–310 mg/100 g reported by Idris^28^, and the calcium content for that of carrot pulp, 34.03 mg/100 g, was observed to have almost similar in value with the result recorded by USDA^35^, 33.00 mg/100 g.

### Anti-nutritional factors, ferric-ion reducing power (FRAP) and total carotenoid contents of raw materials

The anti-nutritional factors FRAP and the total carotenoid analysis results for tef grain, sorghum flour, and carrot pulp are given in Table 4. The result for the condensed tannin content of tef was observed to be almost insignificant (0. 001 mg/100 g), which is in perfect agreement with the result reported by Bultosa^27^, who stated that it is doubtful whether white tef contains condensed tannins. The condensed tannin in sorghum was found to be 4.04 mg/100 g (% as catechin equivalent). Previous studies found that sorghum tannin content ranged from 10 to 2,056 mg/100, 20 to 190 mg/100 g, and 0 to 1,310 mg/100 g tannin as catechin equivalents^42^. The investigation revealed that the condensed tannin content found in this study was in agreement with the reported range of 0 to 1310 mg/100 g^43^. The tef flour phytic acid content, 9.60 mg/100 g, was evaluated to be greater than the result reported by Abebe et al.^40^, 8.42 mg/100 g.

**Table 4:** Anti-nutritional factors, FRAP and total carotenoid contents of raw materials.

Raw materials	Phytic acid (mg/100 g)	Condensed tannin (mg/100 g)	Total phenolics (mg/100 g)	FARP (µmol/100 g)
Tef	9.60 ± 0.48^a^	0.01 ± 0.16^a^	228.78 ± 3.49^a^	117.16 ± 11.48^b^
Sorghum	110.76 ± 1.35^b^	4.04 ± 1.36^a^	371.47 ± 4.31^a^	87.22 ± 10.04^a^
Carrot	ND	ND	170.09 ± 2.44^a^	128.66 ± 2.95^a^

*ND* not detected.

^a,b,c,d,e,f,g^Means ± standard deviations with different letters after data within a column represent differences at 95% probability levels.

The current research outcome on the total phenolic content of sorghum grain was closely in accord with Yang^44^, who reported that the total phenolic content of non-tannin sorghums ranged from 90 to 1820 mg gallic acid equivalent (GAE)/100 g sample and greater than the one reported by Glennie, C. W, which is reported to range from 80 to 100 mg/100 g^45^,. The total carotenoid content for fresh carrot pulp in this study was found to be 8920.67 µg/100 g.

### Effect of fermentation conditions and blending ratio on the proximate composition of dried *injera* products

The main effects of fermentation and blending ratio on the proximate analysis result of fortified *injera* are summarized in Table 5. The moisture content of blended *injera* was observed to vary significantly (*p* < 0.05) due to the effect of fermentation conditions and blending ratios^46^. The highest moisture content value (4.02%) was obtained in the *injera* made by the addition of carrot pulp after fermentation of the tef-sorghum composite flour batter of B_2_ (45% tef + 45% sorghum + 10% carrot), and the lowest result (3.32%) was recorded in the cofermented *injera* blend of B_3_ (60% tef + 30% sorghum + 10% carrot pulp). The result was lightly lower than the moisture content reported by Tiruneh et al.^22^, 4.61 ± 0.07%, which might be due to the higher amount of carrot pulp used for blending purposes, 15%, where the remaining 85% composition was tef flour.

**Table 5:** Effect of fermentation conditions and blending ratio on the proximate composition of fresh dried *injera* products.

Treatment	Energy (kca/g)	Moisture (%)	Ash (%)	Cured protein (%)	Crude fat (%)	Cured fiber (%)	COH (%)	TA	pH
AF*B_1_	378.14 ± 0.97^b^	3.82 ± 0.35^ab^	2.13 ± 0.19^a^	9.77 ± 1.83^b^	1.89 ± 0.05^c^	2.82 ± 0.01^a^	79.00 ± 2.15^a^	3.70 ± 0.00^a^	4.91 ± 0.00^a^
AF*B_2_	375.25 ± 2.30^ab^	4.02 ± 0.55^a^	1.94 ± 0.06^a^	10.18 ± 0.35^b^	1.86 ± 0.05^c^	2.77 ± 0.02^a^	79.42 ± 0.35^a^	2.00 ± 0.00^b^	4.59 ± 0.00^b^
AF*B_3_	374.12 ± 0.72^ab^	3.92 ± 0.39^ab^	1.89 ± 0.03^a^	10.14 ± 1.11^b^	1.88 ± 0.21^c^	2.71 ± 0.05^a^	79.31 ± 1.25^a^	2.40 ± 0.00^c^	4.75 ± 0.00^c^
CF*B_1_	372.70 ± 1.50^a^	3.72 ± 0.38^ab^	2.40 ± 0.52^a^	10.93 ± 1.24^a^	2.87 ± 0.08^a^	2.80 ± 0.79^a^	77.28 ± 1.09^b^	3.60 ± 0.00^d^	3.87 ± 0.00^d^
CF*B_2_	376.01 ± 3.83^ab^	3.71 ± 0.01^ab^	2.31 ± 0.17^a^	11.48 ± 0.70^a^	2.46 ± 0.04^b^	2.72 ± 0.82^a^	76.86 ± 1.44^b^	3.00 ± 0.00^e^	3.86 ± 0.00^e^
CF*B_3_	373.81 ± 2.14^ab^	3.32 ± 0.15^b^	2.20 ± 0.10^a^	11.60 ± 0.45^a^	2.53 ± 0.04^b^	2.52 ± 0.55^a^	76.28 ± 0.67^b^	4.00 ± 0.00^f^	3.81 ± 0.01^f^
Cont.	369.80 ± 1.40^c^	3.48 ± 0.11^c^	1.80 ± 0.07^b^	10.91 ± 0.83^a^	1.87 ± 0.06^c^	2.24 ± 0.12^b^	74.10 ± 0.89^c^	3.30 ± 0.00^e^	4.66 ± 0.00^ g^
Mean	373.88	3.98	2.16	11.35	1.69	2.48	78.31	3.12	4.30
CV	0.58	5.81	4.19	9.46	5.84	5.79	1.65	0.10	0.10

*CV* coefficient of variance, *CF* and *AF* *injera* prepared by addition of carrot pulp before (co-fermented) and after fermentation of tef-sorghum dough, respectively, *cont.* control sample (100% pure tef *injera*), *B*_*1*_ 30% tef + 60% sorghum + 10% carrot pulp; *B*_*2*_ 45% tef + 45% sorghum + 10% carrot; *B*_*3*_ 60% tef + 30% sorghum + 10%carrot), AF*B and CF*B *injera* baked by addition of carrot pulp after fermentation of tef-sorghum blend dough and *injera* made from co-fermented dough interact with blending ratios, respectively.

^a,^^b,c,d,e,f,g^Means ± standard deviations with different letters after data within a column represent differences at 95% probability levels.

The fermentation conditions and blending ratio did not show a considerable effect on the ash content of dried *injera* products^46,47^. Varrying fermentation and blending ratio showed a significant (*p* < 0.05) difference in the protein content of blended *injera* products^46,47^. The highest crude protein content (11.60%) was obtained in B3 (60% tef + 30% sorghum + 10% carrot pulp) of cofermented *injera* product, and the lowest value (9.77%) was obtained in B1 (30% tef + 60% sorghum + 10% carrot) of *injera* prepared by addition of carrot pulp after fermentation of tef-sorghum composite flour batter *injera* sample^22^.

The ratios of mixing and the conditions of fermentation showed a significant (*p* < 0.05) effect on the crude fat content of blended *injera* products^46,48^. The highest result (2.87%) was obtained in B_2_ (45% tef + 45% sorghum + 10% carrot pulp) of co-fermented *injera* product, and the lowest value of (1.86%) was recorded in B_2_ (45% tef + 45% sorghum + 10% carrot pulp) of *injera* prepared from carrot added after fermentation of tef-sorghum composite flour batter^47^, which has an indication that cofermenting of carrot positively affects the moisture content of *injera*. Varrying blending ratios and fermentation conditions exhibited an insignificant (*p* > 0.05) effect on the crude fiber content of fortified *injera* products; however, the carbohydrate content was significantly (*p* < 0.05) influenced by the fermentation conditions and blending ratio, where the highest value of 79.42% was recorded in B2 (45% tef + 45% sorghum + 10% carrot pulp) of *injera* prepared by the addition of carrot into fermented tef-sorghum composite batter, and the lowest value (74.10%) was recorded in the control unblended (100% pure tef) *injera* sample^22,47^.

Additionally, it was noted that the energy content of the blended *injera* was significantly (*p* < 0.05) affected by the fermentation conditions and blending ratio, where the highest value of (378 kcal/100 g) was recorded in B1 (30% tef + 60% sorghum + 10% carrot pulp) of *injera* prepared by the addition of carrot into fermented tef-sorghum composite flour batter, and the lowest value of (369.80 kcal/100 g) was observed in the control (100% pure tef) *injera* sample^47^, showing sorghum to have better energy content than tef flour. Furthermor, the titratable acidity of dried *injera* products and the pH value of fresh dried *injera* products were significantly affected (*p* < 0.05) by fermentation conditions and blending ratios^49^, in which the highest value (4.00) was recorded in B_3_ of co-fermented fresh dried *injera* product and the lowest value (2.00) was obtained in B_2_ *injera*, which was prepared by the addition of carrot pulp into fermented tef-sorghum blended flour batter. This shows the potential of cofermenting to affect the titrable acidity of the freshly dried *injera*. The highest pH value of 4.91 was obtained in B_1_ of *injera*, which was made by the addition of carrot pulp after fermentation of the tef-sorghum blend batter just before baking, and a higher acid pH value of 3.81 was recorded in B_1_ of the co-fermented fresh dried *injera* product. The pH value was observed to favor an increase in the shelf life of the fermented *injera* product^50^.

### Effect of fermentation conditions and blending ratio on the mineral content of fresh dried fortified *injera* products

The effect of fermentation conditions and blending ratios was found to have an insignificant (*p* > 0.05) effect on the phytic acid content and condensed tannin contents^32^ of fortified *injera* products, as depicted in Table 6. However, the phytic acid and condensed tannin contents of 0.52 mg/g and 71 mg/100 g, respectively, were observed to be lower in the control (100% pure tef) sample than in the blended *injera* samples. In contrast, the total phenolic content of the fortified *injera* product was determined to be significantly affected (*p* < 0.05) by fermentation conditions and blending ratios, where the highest value of 133.17 mg/g was obtained in B_1_ (30% tef + 60% sorghum + 10% carrot) of the *injera* prepared by the addition of carrot into the fermented tef-sorghum blended flour batter, and the lowest value of 125.51 mg/g was recorded in B_3_ (60% tef, 30% sorghum + 10% carrot) of the *injera* samples.

**Table 6:** Effect of fermentation conditions and blending ratio on the mineral content of fresh dried *injera* products.

Treatment	Phytic acid mg/100 g	Condensed tannin (mg/100 g)	Total phenolics (mg/100 g)	Fe (mg/100 g)	Zn (mg/100 g)	Ca (mg/100 g)	P (mg/100 g)	FRAP µmol/g	Total carotenoid (µg/100 g)
AF*B_1_	1.25 ± 0.64^a^	1.10 ± 0.13^a^	133.17 ± 0.26^a^	27.26 ± 1.43^a^	2.17 ± 0.50^a^	160.47 ± 4.50^a^	171.27 ± 1.73^a^	47.56 ± 0.56^a^	4122.67 ± 3.06^ba^
AF*B_2_	1.14 ± 0.36^a^	1.11 ± 0.29^a^	133.06 ± 2.51^a^	28.09 ± 2.20^ac^	2.40 ± 0.23^a^	172.53 ± 5.97^b^	170.73 ± 0.91^a^	48.67 ± 0.22^a^	4118.00 ± 2.51^b^
AF*B_3_	0.69 ± 0.18^a^	1.11 ± 0.20^a^	133.51 ± 2.75^a^	33.80 ± 1.41^b^	2.41 ± 0.56^a^	176.72 ± 2.61^c^	171.65 ± 2.06^a^	52.65 ± 5.73^a^	4127.33 ± 2.80^ba^
CF*B_1_	0.99 ± 0.31^a^	0.85 ± 0.05^a^	128.81 ± 2.68^b^	28.92 ± 1.10^c^	2.89 ± 0.10^b^	171.79 ± 4.01^b^	162.44 ± 6.12^b^	48.01 ± 1.43^a^	4219.33 ± 1.81^ba^
CF*B_2_	0.87 ± 0.12^a^	0.88 ± 0.02a	127.55 ± 1.08^b^	31.36 ± 2.20^cb^	2.91 ± 0.07^b^	173.40 ± 6.67^b^	166.79 ± 3.11^ab^	47.88 ± 2.54^a^	4128.33 ± 0.50^a^
CF*B_3_	0.86 ± 0.04^b^	0.86 ± 0.02^a^	125.51 ± 0.88^b^	38.63 ± 0.97^d^	2.95 ± 0.15^b^	186.37 ± 7.93^c^	166.02 ± 5.87^ab^	48.44 ± 1.65^a^	4122.67 ± 3.06^ba^
Cont.	0.52 ± 0.17^c^	0.71 ± 3.06^c^	133.11 ± 3.55^a^	36.79 ± 1.92^e^	2.88 ± 0.92^b^	185.94 ± 5.98^c^	174.49 ± 4.55^c^	38.58 ± 1.14^b^	_ND_
Mean	0.83	0.98	130.56	31.34	2.59	173.38	168.15	48.87	4122.56
CV	3.20	5.72	1.67	6.18	10.08	3.30	2.30	5.57	0.13 cd

*CV* coefficient of variance, *CF* and *AF injera* prepared by addition of carrot pulp before (co-fermented) and after fermentation of tef-sorghum dough, respectively, *cont. q* control sample (100% pure tef *injera*), *B*_*1*_ 30% tef + 60% sorghum + 10% carrot pulp; *B*_*2*_ 45% tef + 45% sorghum + 10% carrot; *B*_*3*_ 60% tef + 30% sorghum + 10%carrot), AF*B and CF*B injera baked by the addition of carrot pulp after fermentation of tef-sorghum blend dough and *injera* made from co-fermented dough interact with blending ratios.

^a,b,c,d^Means ± standard deviations with different letters after data within a column represent differences at 95% probability levels.

The iron contents of blended *injera* products (Table 6) were determined to be positively affected by fermentation conditions and blending ratios, where the highest Fe content (38.63 mg/100 g) was recorded in B_3_ (60% tef + 30% sorghum + 10% carrot pulp) of co-fermented *injera* product and the lowest content (27.26 mg/100 g) was observed in B_1_ (30% tef + 60% sorghum + 10% carrot pulp) of *injera* prepared by the addition of carrot to fermented tef-sorghum batter. This was probably due to the hydrolysis of anti-nutritional (phytic acid and condensed tannin) factors that trap mineral elements, which shows that the iron content was directly related to the proportion of *tef* flour.

In a similar manner, the effect of fermentation condition and blending ratio was observed to have a considerable effect on the Ca content of *the injera* product, showing a significant increase from B_1_ (30% tef + 60% sorghum + 10% carrot pulp) to B_3_ (60% tef + 30% sorghum + 10% carrot pulp) of the fortified *injera* product, where the highest value (186 mg/100 g) was recorded in B_3_ of co-fermented fortified *injera* and the lowest value (160 mg/100 g) was obtained in B_1_ of *injera* prepared by the addition of carrot pulp into fermented tef-sorghum composite flour batter.

### Effect of fermentation conditions and blending ratio on the FRA and carotenoid content of tef-*sorghum*-carrot pulp fortified *injera*

The ferric-reducing antioxidant power (FRAP) was observed to be insignificantly affected by the fermentation conditions and blending ratios^51,52^, where the minimum value of 38.58 µmol/g was recorded in the control (100% pure tef) *injera* sample. However, varying fermentation conditions and blending ratios showed a considerable (*p* < 0.05) effect on the total carotenoid content of the fortified *injera* sample. The highest total carotenoid content (4128.33 µm/g) was recorded in B_2_ of the co-fermented *injera* sample, and the lowest result (4118.00 µg/g) was recorded in B_2_ in *injera* prepared by the addition of carrot pulp after fermentation of the tef-sorghum composite flour batter.

### Effect of fermentation conditions and blending ratio on the sensory quality of tef-*sorghum*-carrot pulp fortified* injera*

**Figure 3:**
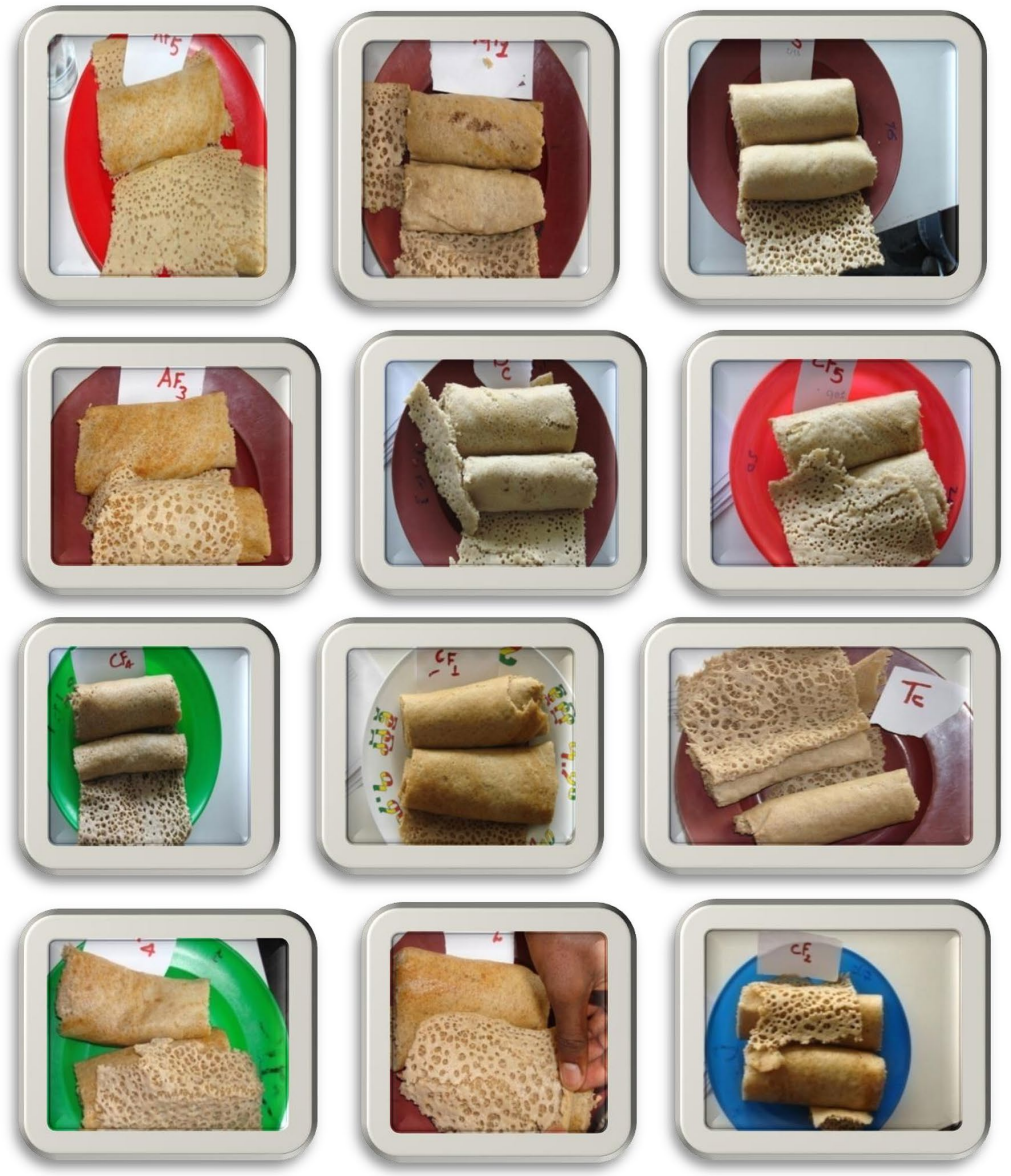
Sensory analysis of *injera* produced from different combination ratios and fermentation conditions.

Table 7 presents the tabulated data showing the significant (*p* < 0.05) effect arising from the fermentation conditions and blending ratios on the color of tef-sorghum-carrot blended *injeras*^53,54^. The findings of the research showed that the color of *injera* made from mixing carrot pulp into fermented tef-sorghum composite flour batter with a blending ratio of B_3_ (60% tef + 30% sorghum + 10% carrot pulp) was thought to be better preferred by the panelists, while *injera* prepared from co-fermented B_1_ (30% tef + 60% sorghum + 10% carrot) was discovered to be the least preferred color. A color value with a maximum value of 6.36 was recorded in the *injera* produced by adding carrot pulp to the tef-sorghum composite flour batter after it was fermented, as in B_3_ (60% tef + 30% sorghum + 10% carrot pulp), and the lowest score (5.33) was obtained in the *injera* prepared from the co-fermented batter at a blending ratio of 30% tef: 60% sorghum: 10% carrot pulp. *Injera* produced from different blending rations and fermentation conditions is shown in Fig. 3.

**Table 7:** Effect of fermentation and blending ratio on the sensory quality of freshly baked *injera*.

Treatment	Color	Oder	Flavor	Sour character	*Injera* eyes	*Injera* underneath	*Injera* Texture	Overall acceptance
AF*B_1_	6.00 ± 0.00^c^	5.27 ± 0.27^e^	5.14 ± 0.50^c^	5.26 ± 0.44^c^	4.98 ± 0.68^d^	4.56 ± 0.67^d^	4.70 ± 0.46^e^	4.90 ± 0.36^d^
AF*B_2_	6.20 ± 0.45^c^	5.50 ± 0.00^dc^	5.03 ± 0.29^c^	5.16 ± 0.37^c^	5.14 ± 0.57^d^	5.02 ± 0.91^ cd^	5.02 ± 0.55^d^	5.39 ± 0.50^c^
AF*B_3_	6.54 ± 0.58^a^	5.43 ± 0.50^d^	6.33 ± 0.95^a^	5.49 ± 0.50^b^	6.08 ± 0.93^b^	5.92 ± 0.63^b^	5.43 ± 0.54^c^	5.41 ± 0.50^c^
CF*B_1_	5.35 ± 0.48^d^	6.00 ± 0.00^b^	6.00 ± 0.00^b^	5.98 ± 0.14^a^	5.22 ± 0.46^d^	5.27 ± 0.45^c^	5.29 ± 0.70^c^	5.51 ± 0.38^c^
CF*B_2_	5.33 ± 0.51^d^	5.65 ± 0.34^c^	5.96 ± 0.19^b^	6.00 ± 0.71^a^	6.00 ± 0.00^c^	5.71 ± 0.46^bc^	5.77 ± 0.43^b^	5.79 ± 0.41^b^
CF*B_3_	6.36 ± 0.78^b^	6.32 ± 0.57^a^	6.38 ± 0.49^a^	6.14 ± 0.53^a^	6.50 ± 0.68^a^	6.72 ± 0.40^a^	6.78 ± 0.33^a^	6.58 ± 0.41^a^
Cont.	6.07 ± 0.71^e^	6.03 ± 0.80^e^	6.26 ± 0.65^f^	6.01 ± 0.45^f^	6.95 ± 0.68^e^	6.71 ± 6.15^a^	6.80 ± 0.51^a^	6.11 ± 0.40^b^
Mean	5.21	5.17	5.57	5.44	5.36	5.12	5.51	5.38
CV	8.97	4.44	7.52	6.38	9.61	6.43	4.53	6.04

*CV* coefficient of variance, *CF* and *AF injera* prepared by addition of carrot pulp before (co-fermented) and after fermentation of tef-sorghum dough, respectively, *cont. q* control sample (100% pure tef *injera*), *B*_*1*_ 30% tef + 60% sorghum + 10% carrot pulp; *B*_*2*_ 45% tef + 45% sorghum + 10% carrot; *B*_*3*_ 60% tef + 30% sorghum + 10%carrot), AF*B and CF*B injera baked by the addition of carrot pulp after fermentation of tef-sorghum blend dough and *injera* made from co-fermented dough interact with blending ratios.

^a,b,c,d,e^Means ± standard deviations with different letters after data within a column represent differences at 95% probability levels.

The *injera* flavor was observed to be significantly (*p* < 0.05) influenced by the combined effect of the blending ratio and fermentation conditions^48,55^, as shown in Table**,** in which the co-fermented *injera* prepared from the blending ratio of B_3_ was seen to have the highest value of (6.32) odor sense, whereas the *injera* made from the inclusion of carrot pulp after fermentation of the tef-sorghum composite flour batter had the lowest value (5.27). Similarly, a co-fermented blend of B_3_ was found to have the best flavor value (6.38) for fortified *injera*, while B_1_, which included carrot pulp after the tef-sorghum composite flour batter was fermented, had the lowest flavor value (5.14).

It was found that the combined effect of the blending ratio and the fermentation condition was observed to show a significant (*p* < 0.05) difference on the *injera* eyes of fortified *injera* products, where the control (100% tef) *injera* sample was seen to have the highest result (6.95), while B_1_ of the *injera* made by adding carrot pulp after the tef-sorghum composite flour batter fermented had the lowest value (4.98). The texture of the *injera* was also observed to be affected by fermentation conditions and blending ratio, where the *injera* made by adding carrot pulp to a batter made of fermented tef and sorghum flour was found to have the lowest value of 4.70, and the highest value of (6.80) was discovered in the control (100% tef) *injera* sample. Similarly, the overall acceptance of fortified *injera* products was evaluated to be affected by the combined effect of fermentation conditions and blending ratios, wherein the *injera* made by adding carrot pulp to the fermented tef-sorghum flour composite batter in B_1_ was evaluated to have the lowest result (4.90) and the highest value of 6.58 was found in the co-fermented *injera* product B_3_.

### Effect of fermentation conditions and blending ratio on microbial count of fresh *injera*

Table 8 shows that the microbial composition of blended *injera* products made from tef, sorghum, and carrot pulp varied significantly (*p* < 0.05) depending on the varrying blending ratios and the fermentation conditions^46,55^. Due to the varrying fermentation condtions and blending ratios, the yeast-mold count for the day 2 log showed a significant (*p* < 0.05) difference in blended *injera* products, wherein B1 of the *injera* produced by adding carrot pulp after the tef-sorghum composite flour batter fermented was evaluated to have the highest count of (3.82 cfu/g), and B3 of the co-fermented *injera* product had the lowest count (2.65 cfu/g). Likewise, the yeast-mold count of the day 4 log of baked *injera* and day 6 baked *injera* sample were also observed to be significantly (*p* < 0.05) influenced by the blending ratio and the fermentation conditions, in which the B_1_ *injera* prepared by the addition of carrot pulp after fermentation of tef-sorghum blended flour batter was evaluated to have a higher value of (3.97 cfu/g) yeast-mold count and the co-fermented *injera* product of B_3_ was seen to have the lowest count of (2.17 cfu/g) for the day 4 log baked *injera*.

**Table 8:** Effect of fermentation conditions and blending ratios on microbial count of fresh *injera*.

Treatment	Yeast –mold count			Total plate count			Coli forms		
	Day 2 log (cfu/g)	Day 4 log (cfu/g)	Day 6 log (cfu/g)	Day 2 log (cfu/g)	Day 4 log (cfu/g)	Day 6 log (cfu/g)	Day2 log (cfu/g)	Day 4 log (cfu/g)	Day 6 log (cfu/g)
AF*B_1_	3.82 ± 0.07^c^	3.97 ± 0.17^a^	4.04 ± 0.05^ab^	2.49 ± 0.11^ab^	3.52 ± 0.31^a^	3.77 ± 0.23^a^	ND	ND	3.29 ± 0.16^a^
AF*B_2_	2.66 ± 0.01^b^	3.61 ± 0.11^b^	4.03 ± 0.40^ab^	2.54 ± 0.00^b^	3.04 ± 0.08^b^	3.73 ± 0.18^a^	ND	ND	3.03 ± 0.08^b^
AF*B_3_	2.91 ± 0.00^d^	3.19 ± 0.06^c^	3.90 ± 0.01^a^	2.39 ± 0.07^a^	2.99 ± 0.19^b^	3.77 ± 0.12^a^	ND	ND	2.86 ± 0.16^b^
CF*B_1_	2.95 ± 0.00^a^	3.41 ± 0.02^d^	4.01 ± 0.03^ab^	2.49 ± 0.05^ab^	3.18 ± 0.17^b^	3.83 ± 0.14^a^	ND	ND	2.57 ± 0.11^c^
CF*B_2_	2.83 ± 0.01^a^	3.25 ± 0.04^c^	3.87 ± 0.01^a^	2.48 ± 0.02^ab^	2.65 ± 0.09^c^	4.01 ± 0.10^a^	ND	ND	3.10 ± 0.19^bc^
CF*B_3_	2.65 ± 0.04^b^	3.17 ± 0.02^c^	3.88 ± 0.00^a^	2.27 ± 0.01^c^	2.63 ± 0.01^c^	3.89 ± 0.05^a^	ND	ND	2.92 ± 0.07^b^
Cont.	2.98 ± 0.03^a^	4.03 ± 0.06^a^	4.07 ± 0.06^b^	2.95 ± 0.06^d^	4.01 ± 0.02^d^	4.06 ± 0.05^a^	ND	ND	3.60 ± 0.19^d^
CV	1.08	2.51	4.12	2.32	5.66	3.84	–	–	4.55

*CV* coefficient of variance, *CF* and *AF injera* prepared by addition of carrot pulp before (co-fermented) and after fermentation of tef-sorghum dough, respectively, *Cont.* control sample (100% pure tef *injera*), *ND* not detected, *B*_*1*_ 30% tef + 60% sorghum + 10% carrot pulp; *B*_*2*_ 45% tef + 45% sorghum + 10% carrot; *B*_*3*_ 60% tef + 30% sorghum + 10%carrot), AF*B and CF*B injera baked by the addition of carrot pulp after fermentation of tef-sorghum blend dough and *injera* made from co-fermented dough interact with blending ratios, respectively.

^a,b,c,d^values are means ± standard deviations with different letters after data within a column represents differences at 95% probability levels.

On the other hand, for the day 6 baked enjera, a better maximum count of 4.07 cfu/g was observed in the control (100% pure tef) *injera* sample, while there were negligible variations between the blended *injera* samples. The plate count and coliform count of blended baked *injera* samples were also observed to be affected by the combined effect of fermentation conditions and bleding ratios, where the highest plate count (2.54 cfu) for the day 2 log was recorded in B_2_ of *injera* prepared by the addition of carrot pulp after fermentation of tef-sorghum composite flour batter and the lowest (2.27 cfu/g) count was recorded in B_3_ of co-fermented *injera* sample. Similarly, for the plate count of day 4 blended *injera* samples, the highest count of 3.52 cfu/g was obtained in *injera* prepared by the addition of carrot pulp after fermentation of the tef–sorghum blended flour batter, and a lower plate count of 2.63 cfu/g was recorded in B3 of the co-fermented *injera* sample. As depicted in Table 8 the day 6 log coliform count, the highest count of 3.29 cfu/g was obtained in B1 of *injera* prepared by the addition of carrot pulp into fermented tef-sorghum blended flour batter, and the lowest count of 2.57 cfu/g was recorded in B_1_ of co-fermented *injera* sample. However, for the day 2 and day 4 logs, insignificant numbers of coliform counts were recorded (ND).

## Conclusion

This study has proven the possibility of making nutritionally acceptable *injera* by evaluating the effect of sorghum and carrot pulp blending ratios under various fermentation conditions on the nutritional and sensory quality of tef *injera*. According to the findings of the study, the sorghum and carrot pulp blending ratios were observed to have a major impact on the nutritional values and sensory quality of the *injera*. Furthermore, the fermentation conditions were also found to have a significant effect on the tef-sorghum-carrot fortified *injera* product quality, affecting the nutritional, anti-nutritional, microbial content and sensory properties of the *injera product*. Co-fermentation of tef-sorghum-carrot pulp was found to increase crude protein, crude fat, titratable acidity, iron, zinc and calcium but was evaluated to reduce moisture content, crude fiber content phosphorus content, carbohydrate, ash, energy, pH, microbial count, phytic acid and condensed tannin content. The *injera* product made using the B_3_ blend of 60% tef + 30% + 10% carrot pulp showed the highest sensory scores, crude protein, crude fat, TTA, Fe, Ca and FRAP and lowest energy content, lowest microbial counts and phytic acid content. On the other hand, the *injera* produced using the blending ratio of B_1_ (30% tef + 60 sorghum + 10% carrot pulp) showed the lowest crude protein, total phenolics, Ca, and P and the highest moisture, ash, crude fiber, pH, TTA, phytic acid and Fe contents. It was recommended that blending -cereal-based functional foods with carrots should be investigated further to improve.

Vitamin A deficiency in *injera* products, where carrots are recommended to be included in a daily diet plan to improve vitamin A content in the *injera*, and this seems to be a promising approach since the *injera* is the national staple food for more than 80% of Ethiopians. Starch pasting properties and protein and starch digestibility of tef-sorghum-carrot pulp fortified *injera* should be studied. It is also recommended that processing and storage of foods should be optimized to prevent carotenoid losses. Additionally, further research should be done on the effect of the sorghum blending ratio and carrot pulp supplementation on the water activity and viscosity of tef dough. here researchers will be advised not to neglect the existing traditional food technologies for significant changes to be made in Ethiopian food systems. Finally, the study recommends the blending ratio B3 (60%tef: 30%sorghum:10% carrot) of co-fermented *injera* product for practical application of commercializing the product due to its very good nutritional improvement.

## Data Availability

The authors confirm that the data supporting the findings of this study are available within the article.

## References

[1] Fekadu T, Cassano A, Angós I, Maté JI (2022). Effect of fortification with eggshell powder on *injera* quality. LWT.

[2] Mengesha Y, Tebeje A, Tilahun B (2022). A review on factors influencing the fermentation process of teff (*Eragrostis teff*) and other cereal-based Ethiopian *injera*. Int. J. Food Sci..

[3] Han JA, Kwon KH (2022). Development potential of *Eragrostis tef* as a flour alterntive. Carpathian J. Food Sci. Technol..

[4] Barretto R (2021). Teff (*Eragrostis tef*) processing, utilization and future opportunities: A review. Int. J. Food Sci. Technol..

[5] El Shafey AM (2020). Green synthesis of metal and metal oxide nanoparticles from plant leaf extracts and their applications: A review. Green Process. Synth..

[6] Hegde VS, Tripathi S, Bharadwaj C, Agrawal PK, Choudhary AK (2018). Genetics and genomics approaches to enhance adaptation and yield of chickpea (*Cicer arietinum* L.) in semi-arid environments. SABRAO J. Breed. Genet..

[7] Elhassan MSM, Emmambux MN, Hays DB, Peterson GC, Taylor JRN (2015). Novel biofortified sorghum lines with combined waxy (high amylopectin) starch and high protein digestibility traits: Effects on endosperm and flour properties. J. Cereal Sci..

[8] Visarada KBRS, Aruna C, Tonapi VA, Talwar HS, Are AK, Venkatesh Bhat B, Ravinder Reddy CH, Dalton TJ (2020). Breeding sorghum for specific end uses. Sorghum in the 21st century: Food–fodder–feed–fuel for a rapidly changing world.

[9] Manzoor M, Shams R, Rizvi QEH, Dar AH, Singh A, Mir SA, Shah MA, Hamdani AM (2021). Structural aspects of gluten free breads. Gluten-free bread technology.

[10] Acharya, D. Preparation and quality evaluation of malted sorghum incorporated bread. (2021).

[11] Yetneberk S, Rooney LW, Taylor JRN (2005). Improving the quality of sorghum *injera* by decortication and compositing with tef. J. Sci. Food Agric..

[12] Ari AP, Demirkesen I, Bean SR, Aramouni F, Boyaci IH (2022). Sorghum flour application in bread: Technological challenges and opportunities. Foods.

[13] Yetneberk S, De Kock HL, Rooney LW, Taylor JRN (2004). Effects of sorghum cultivar on *injera* quality. Cereal Chem..

[14] Mezgebe, A. G. Sorghum waxy and high protein digestibility traits and their relationship with malting and dough-based product making quality. 1–183 (2018).

[15] Connolly EL (2021). Glucosinolates from cruciferous vegetables and their potential role in chronic disease: Investigating the preclinical and clinical evidence. Front. Pharmacol..

[16] Dary O, Hurrell R (2006). Guidelines on food fortification with micronutrients. World Health Organ. Food Agric. Organ. United Nations Geneva, Switz..

[17] Majerska J, Michalska A, Figiel A (2019). A review of new directions in managing fruit and vegetable processing by-products. Trends Food Sci. Technol..

[18] Muhammad, H. F. L. & Dickinson, K. M. Nutrients, energy values and health impact of conventional beverages. in *Nutrients in Beverages* 41–75 (Elsevier, 2019).

[19] Reyes CM, Cornelis MC (2018). Caffeine in the diet: Country-level consumption and guidelines. Nutrients.

[20] Prieciņa, L. & Kārkliņa, D. Influence of steam treatment and drying on carrots composition and concentration of phenolics, organic acids and carotenoids. in *Proceedings of the Latvian Academy of Sciences. Section B. Natural, Exact, and Applied Sciences.* **72**, 103–112 (2018).

[21] Upadhyay, S. Strategy and approaches of extraction of natural bioactive compounds and secondary metabolites from plant sources. *Bioact. Components A Sustain. Syst. Good Heal. Well-Being* 423–438 (2022).

[22] Tiruneh AT, Bultosa G, Zewdie TA, Abera AA (2020). Effect of mango and carrot fortification on proximate composition, β-carotene and sensory properties of teff *injera*. Cogent Food Agric..

[23] Amoroso L (2021). Sustainable cellulose nanofiber films from carrot pomace as sprayable coatings for food packaging applications. ACS Sustain. Chem. Eng..

[24] Do Nascimento KDO, Paes S, de Oliveira IR, Reis IP, Augusta IM (2018). Teff: Suitability for different food applications and as a raw material of gluten-free, a literature review. J. Food Nutr. Res..

[25] Nguyen TTT (2007). Effect of fermentation by amylolytic lactic acid bacteria, in process combinations, on characteristics of rice/soybean slurries: A new method for preparing high energy density complementary foods for young children. Food Chem..

[26] Aggarwal P, Kaur R (2015). Development of intermediate moisture product from carrot pulp. Veg. Sci..

[27] Bultosa G (2007). Physicochemical characteristics of grain and flour in 13 tef [*Eragrostis tef* (Zucc.) Trotter] grain varieties. J. Appl. Sci. Res..

[28] Idris WH, Abdel Rahaman SM, ElMaki HB, Babiker EE, El Tinay AH (2007). Effect of malt pretreatment on HCl extractability of calcium, phosphorus and iron of sorghum (*Sorghum biocolor*) cultivars. Int. J. food Sci. Technol..

[29] Wondimu, A. & Tekabe, F. Utilization of tef in the Ethiopian diet. in *Proceedings of the International Workshop on Tef Genetics and Improvement, Debre Zeit* (2001).

[30] Minweyelet M, Solomon WK, Bultosa G (2021). Effects of extrusion operating conditions and blend proportion on the physicochemical and sensory properties of teff-rice blend extruded products. Food Res..

[31] Committee, A. A. of C. C. A. M. *Approved methods of the American association of cereal chemists*. vol. 1 (Amer Assn of Cereal Chemists, 2000).

[32] Leykun T, Admasu S, Abera S (2020). Evaluation of the mineral content, phyto-chemicals profile and microbial quality of tef *injera* supplemented by fenugreek flour. J. Food Sci. Technol..

[33] Steel, R. G. D. Analysis of variance II: Multiway classifications. *Princ. Proced. Stat. A Biometrical Approach* 204–252 (1997).

[34] Socioeconomics, I. C. R. I. for the S.-A. T. *et al. The world sorghum and millet economies: Facts, trends and outlook*. (Food & Agriculture Org., 1996).

[35] USDA. Composition of Foods: USDA National Nutrient Database for Standard Reference, Legacy (2018). *U.S. Dep. Agric. Agric. Res. Serv. Beltsv. Hum. Nutr. Res. Cent. Nutr. Data Lab.* **2**, 1–136 (2018).

[36] Kebede, L. Effect of extrusion operating conditions on the physicochemical and sensory properties of grain teff puffed products. (2006).

[37] Dendy, D. A. V. *Sorghum and millets: Chemistry and technology*. (American Association of Cereal Chemists, 1995).

[38] Smith CW, Frederiksen RA (2000). Sorghum: Origin, history, technology, and production.

[39] Bultosa G, Taylor JRN (2004). Paste and gel properties and in vitro digestibility of Tef [*Eragrostis tef* (Zucc.) Trotter] starch. Starch/Staerke.

[40] Abebe Y (2007). Phytate, zinc, iron and calcium content of selected raw and prepared foods consumed in rural Sidama, Southern Ethiopia, and implications for bioavailability. J. Food Compos. Anal..

[41] Haileslassie K, Mulugeta A, Girma M (2013). Feeding practices, nutritional status and associated factors of lactating women in Samre Woreda, South Eastern Zone of Tigray. Ethiopia. Nutr. J..

[42] Chem63_4.Pdf.

[43] Afify AE-MMR, El-Beltagi HS, El-Salam SMA, Omran AA (2012). Biochemical changes in phenols, flavonoids, tannins, vitamin E, β–carotene and antioxidant activity during soaking of three white sorghum varieties. Asian Pac. J. Trop. Biomed..

[44] Yang, L. Chemopreventive potential of sorghum with different phenolic profiles. (2010).

[45] Glennie CW (1983). Polyphenol changes in sorghum grain during malting. J. Agric. Food Chem..

[46] Desalegn, M. Effects of blending ratio and fermentation time on physicochemical and sensory acceptability of *injera* prepared from pumpkin-tef composite flour. (2017).

[47] Mihrete Y, Bultosa G (2017). The effect of blending ratio of tef [*Eragrostis tef* (Zucc) Trotter], sorghum (*Sorghum bicolor* (L.) Moench) and faba bean (Vicia faba) and fermentation time on chemical composition of *injera*. J. Nutr. Food Sci..

[48] Wegari, M. Ms. & Abera, S. Effects of blending ratio and fermentation time on physicochemical and sensory acceptability of teff taro composite flour *injera*. (2022).

[49] Zewdie S, Urga K, Nigatu A (1997). Co-fermentation of kocho with barley for an improved *injera*. SINET Ethiop. J. Sci..

[50] Ampadu, E. W. Soybean (glycine max) processing and performance. (1994).

[51] Gupta S, Jaiswal AK, Abu-Ghannam N (2013). Optimization of fermentation conditions for the utilization of brewing waste to develop a nutraceutical rich liquid product. Ind. Crops Prod..

[52] Najgebauer-Lejko D, Sady M, Grega T, Walczycka M (2011). The impact of tea supplementation on microflora, pH and antioxidant capacity of yoghurt. Int. Dairy J..

[53] Anberbir SM (2023). Effect of blending ratio and fermentation time on the physicochemical, microbiological, and sensory qualities of *injera* from teff, pearl millet, and buckwheat flours. CyTA-J. Food.

[54] Shumoy H, Gabaza M, Vandevelde J, Raes K (2017). Soluble and bound phenolic contents and antioxidant capacity of tef *injera* as affected by traditional fermentation. J. Food Compos. Anal..

[55] Terefe, A. Effect of processing methods of fenugreek seed and blending ratio with sorghum on the nutritional and sensory quality of sorghum *injera*. (2017).

